# MicroRNA-32 promotes calcification in vascular smooth muscle cells: Implications as a novel marker for coronary artery calcification

**DOI:** 10.1371/journal.pone.0174138

**Published:** 2017-03-20

**Authors:** Jianghua Liu, Xinhua Xiao, Yingying Shen, Ling Chen, Canxin Xu, Heng Zhao, Ying Wu, Qinghai Zhang, Jing Zhong, Zhenwang Tang, Changhui Liu, Qiang Zhao, Yi Zheng, Renxian Cao, Xuyu Zu

**Affiliations:** 1 Institute of Clinical Medicine, the First Affiliated Hospital of University of South China, Hengyang, People’s Republic of China (PRC); 2 Department of Metabolism and Endocrinology, the First Affiliated Hospital of University of South China, Hengyang, PRC; 3 Department of Pathology and Immunology, University of Washington School of Medicine, St. Louis, Missouri, United States of America; 4 Department of Cardiovascular Medicine, the First Affiliated Hospital of University of South China, Hengyang, PRC; University of California, Los Angeles, UNITED STATES

## Abstract

Cardiovascular calcification is one of the most severe outcomes associated with cardiovascular disease and often results in significant morbidity and mortality. Previous reports indicated that epigenomic regulation of microRNAs (miRNAs) might play important roles in vascular smooth muscle cell (VSMC) calcification. Here, we identified potential key miRNAs involved in vascular calcification *in vivo* and investigated the role of miR-32-5p (miR-32). According to microarray analysis, we observed increased expression of miR-125b, miR-30a, and miR-32 and decreased expression of miR-29a, miR-210, and miR-320 during the progression of vascularcalcification. Additionally, gain- and loss-of-function studies of miR-32 confirmed promotion of VSMC calcification in mice through the enhanced expression of bonemorphogenetic protein-2, runt-related transcription factor-2(RUNX2), osteopontin, and the bone-specific phosphoprotein matrix GLA protein *in vitro*. Moreover, miR-32 modulated vascularcalcification progression by activating phosphoinositide 3-kinase (PI3K)signaling and increasing RUNX2 expression and phosphorylation by targeting the 3′-untranslated region of phosphatase and tensin homolog Mrna (PTEN) in mouse VSMCs. Furthermore, we detected higher miR-32 levels in plasmafrom patients with coronary artery disease with coronary artery calcification (CAC) as compared with levels observed in non-CAC patients (*P* = 0.016), further confirming miR-32 as a critical modulator and potential diagnostic marker for CAC.

## Introduction

Cardiovascular calcification is a major characteristic of chronic inflammatory disorders, including atherosclerosis, type 2 diabetes mellitus, and chronic kidney disease, and associated with significant morbidity and mortality [1].Vascular calcification is a precisely regulated process sharing similarities with bone mineralization and remodeling, withphenotypic de-differentiation of mature smooth muscle cells into an osteogenic phenotype being a key event in vascular calcification. Alterations in the response of genes, including bone morphogenetic protein-2 (BMP2), runt-related transcription factor-2 (RUNX2), osteopontin (OPN), the bone-specific phosphoprotein matrix GLA protein (MGP), and alkaline phosphatase (ALP), to inflammation, oxidative stress, bone morphogenesis, and high phosphate levelsare involved in the transition from mature smooth muscle cells to an osteogenic phenotype [1–6]. Therefore, an in-depth understanding of the mechanisms underlying the regulation of vascular smooth muscle cell (VSMC)gene expression associated with this phenotype is critical.

An osteoprotegerin knockout (OPG^−/−^)mouse model has been studied for decades, with artery calcification being one of the most significant physiological characteristics observed in OPG^−/−^mice. Simonet *et al*[7] reportedthat arteries exhibiting calcification in OPG^−/−^ mice are sites of endogenous OPG expression, suggesting that OPG might play a role in protecting arteries from pathological calcification.Additionally, postnatal bone loss inOPG^−/−^ mice was associated with active bone remodeling, suggesting that OPG regulates osteoclast development and recruitment[8].Given the close connection betweenOPG and calcification, OPG^−/−^ miceare widely used as a typical calcification model. Min *et al*[9] reportedthat the OPG/OPG ligand/receptor activator of nuclear factor κB-signaling pathway in OPG^−/−^ mice plays a preventative role in vascular calcification.The OPG^−/−^ mouse model was also used as an *in vivo* arterial-calcification model to verify the ameliorative role of omentin-1in calcification [10].Here, we used OPG^−/−^ mice as *in vivo* models to explore the physiological progression of calcification.

MicroRNAs (miRNAs) are a large class of evolutionarily conserved, small, endogenous, noncoding RNAs that function as essential modulators of gene expression.MiRNAsregulate diverse biological and pathological processes, including apoptosis, cell proliferation and differentiation, organogenesis and organ development [11]. In addition to its physiological roles, miRNA dysregulation often leads to impaired cellular function and disease progression [12].Although miRNAs are crucial to VSMC development, proliferation, and function, the central role of miRNAs as essential regulators of the cardiovascular system continues to be investigated [13–16], with recent reports indicating their involvement in cardiovascular calcification [17–20].

In this study, we evaluateddynamic miRNA-expression profiles observed during the progression of vascularcalcification and identifiedpotential key miRNAs involved in this process. Our findings revealed an active role for miR-32 in calcification progression and confirmed that miR-32 promoted vascularcalcification progression, establishing it asa potential diagnostic marker.

## Materials and methods

### Animals

All animal studies were performed according to the NIH guidelines for the care and use of laboratory animals with the approval of the Experimental Animal Committee at University of South China (No.2012-15). OPG-knockout (OPG^−/−^) and wild type (WT) male mice were provided by the Shanghai Research Center for Biomodel Organisms. An OPG-knockout construct was prepared as described by Bucay et al [8]. The PGK-neo cassette replaced a 279-bp region of *OPG* exon 2 that codes for the first 93 amino acids of the mature OPG protein, thus producing OPG^−/−^mice. In this study, OPG^−/−^and WT mice with a mixed C57BL/6J×129/SV background were all bred by heterozygous mating of heterozygous (OPG^+/−^) mice. Prior to the experiments, the animals were identified by performing polymerase chain reaction using DNA from mice tails. At the end of experiments, OPGknockout and WT mice were anaesthetized with 2% isoflurane and the aortas of mice were resected, and subjected to microRNA microarray. All the animals were housed under specific pathogen-free (SPF) conditions (22°C, 12 h/12 h light/dark, 50%–55% humidity) with free access to food pellets and tap water in the experimental animal center of the University of South China.

### Calcified mice vascular smooth muscle cells

The mice vascular smooth muscle cell line (MVSMC) was derived from aortic smooth muscle cells of 6 weeks male C57BL/6J mice according to the protocols described previously[21].Briefly, mice were anaesthetized with 2% isoflurane and the adventitia was removed and the aorta was cut open to expose the endothelial layer. Tissues were pooled for digestion with 1 mg/ml trypsin (Beyotime Institute of Biotechnology, China) to remove remaining adventitia and endothelium. Aortas were then placed in digestion medium contain 425 U/ml collagenase type II (Invitrogen, Carlsbad, CA, USA) until a single cell suspension was obtained. Primary MVSMCs were grown in Dulbecco’s modified Eagle’s medium (DMEM, Gibco BRL, Grand Island, USA) supplemented with 10% heated fetal bovine serum (FBS, Gibco, Australia), 100 U/ml penicillin-streptomycin at 37°C, 5% CO2. MVSMCs were seeded at a density of 1.5×10^4^ cells/cm^2^. At confluence, the calcification of MVSMCs were induced in the presence of calcifying medium containing 2.5 mM β-glycerophosphate (Sigma, Poole, Dorset, UK) and 50μg/ml ascorbic acid (Sigma, Saint Louis, USA) for 21 days. The calcification status was detected by Alizarin Red staining. MVSMCs maintained with normal culture medium were utilized as control cells in the miRNA microarray.

### Calcification determination

Calcified MVSMCs were stained with alizarin red. After washing in distilled water, cultures were fixed with 4% paraformaldehyde in PBS for 15 min, and then stained with 2% alizarin red in H_2_O for 30 min at room temperature. After staining, cultures were washed three times, and the nodules were visualized in the light microscope. For quantification of calcium deposition, MVSMCs were decalcified with 0.6M HCl for 24 h, and the calcium content in the supernatant was determined using a calcium colorimetric assay kit (BioVision, Mountain View, CA, USA).

### Plasma sample preparation

Fresh whole blood from mice (by eyeball removal)or pateints was collected into tubes with anticoagulanttreatment, and then centrifuged at 1000×g for 15 min at 4°C. The supernatant were transferred to clean tubes for further study.

### Transfection

MVSMCs were seeded into 6-well plateswith2×10^5^ cells per well. After overnight incubation, remove the culture medium and wash cells with PBS. The pre-miR32, anti-miR32, or negative control was mixed with lipo2000 vector, and was then added into designatedwells with OPTI-MEM (Invitrogen). After transfection for 6 h, remove the transfection medium, and add enough fresh culture medium without antibioticto continue 4 day incubation.

### Real-time PCR

Based on the differential miRNA profiles during vascular calcification progression *in vivo*, 8 miRNAs and Dicer/Drosha were chosen for the validation using quantitative PCR with SYBR Green qRCR Mix (Toyobo Co.,Ltd, Life Science Department, Osaka, Japan). The programs of this method are as follows: 3 minutes at 95°C; then 10 seconds at 95°C, 30 seconds at 58 or 62°C, 35–40 cycles were performed followed by melting-curve analysis to verify the correctness of the amplicon. To determine whether miR-32 affects the progression of MVSMC calcification, the mimic miR-32 and anti miR-32 (RiboBio Co.Ltd., China) were transfected into MVSMCs, and the transfection efficiency was confirmed by Q-PCR as mentioned. Total RNA from transfected MVSMCs was then isolated and reversely transcribed by use of a reverse transcription system (GenePharma or GeneCopoeia Co.Ltd., China).The amount of PCR product formed in each cycle was evaluated by SYBR Green qRCR Mix (Toyobo, Osaka, Japan) as above. The actin values were used as standard for presentation of the mRNA data of the different transcripts. U6 was used as normalizing miRNA in aortas, and miR-16 was used as circulating miR normalizer in plasma. Because U6 was widely used as an endogenic normalizer for miRNA detection in tissues and cells, but not suitable for circulating miRNAs [22], such as plasma; MiR-16 was found to be steadily expressed in circulating system, and was used to be housekeeping miR for other miRs detection in blood samples [23, 24]. So, U6 or miR-16 was respectively used here as normalizing miRNA in aortas or plasma, because of their steady expression.Analysis of data was performed according to the manufacturer’s instructions using Light Cycler software version 3.5.3 (Roche, Mannheim, Germany). Oligonucleotide primers for PCR were designed and listed in S4 Table.

### Western blot

Detailed procedures were described before [25,26]. Simply, the mimic miR-32 and anti miR-32 transfected MVSMCs containing equal amounts of total protein were resolved by SDS–PAGE and then transferred to a polyvinylidenefluoride membrane (PVDF,Millipore,USA). The membranes were then incubated with anti-PTEN, p-Akt, Akt (ImmunoWay Bio.Co.,Newark, DE, USA), p-RUNX2 and RUNX2(Abzoom, Dallas, TX, USA) for 1 h at room temperature. The membranes were washed three times in TBS with 0.05% Tween 20 (Genview Scientific, China) before incubating for 45 min at room temperature with anti-rabbit or anti-mouse antibody conjugated to horseradish peroxidase(Jackson, West Grove, PA, USA). As an internal control, each membrane was probed with an β-actin antibody (Abzoom, Dallas, TX, USA).The fluorescence signal was then detected by use of the Fluorchem E system (Santa Clara, CA, USA).

### *In situ* hybridization

Aortic arteries paraffin sections of wild-type and OPG^-/-^ mice at 4 and 12 weeks of age fixed in PLP were investigated by nonradioactive *in situ* hybridization technique with digoxigenin-labelled RNA probes[27]. Briefly, paraffin-embedded tissues.were cut into 3mm thin sections and dewaxied.After permeabilisation, sections were prehybridised with 50% formamide in 2×standard sodium citrate for 2h at 37°C. Hybridisation was carried out overnight at 37°C with the appropriate hybridisation mixture containing either the digoxigenin-labelled sense or anti-sense probe. Post-hybridisation involved a series of washes, performed with decreasing concentrations of saline-sodium citrate buffer at 37°C. Samples were washed with ribonuclease solution for 20 min at 37°C to remove any unbound RNA probes. All experiments were done by Universal Fluorescence *in situ* hybridization Detection Kit (Boster Co.Ltd., China). Antisense LNA-modified oligodeoxynucleotide probes targeting mmu-miR-32-5p were designed using mature miRNA sequences from miRBase.Mmu-miR-32-5p probe was as follow: 5’-TGCAACTTAGTAATGTGCAATA- 3’.

### Northern blotting

Total RNAs were extracted from MVSMCs transfected with mimic miR-32 and anti miR-32. Hybridizations were performed as previously described [28]. Total RNAs were size fractionated by gel electrophoresis on 1% agarose/5% formaldehyde gels and transferred to a nylon membrane (Hybond-N+; Amersham, Buckinghamshire, UK) and immobilized with ultraviolet cross-linking. The membrane was pre-hybridized for 15 min at 68°C in QuickHyb Hybridization Solution (Stratagene, USA) with 20mg/mL salmon sperm DNA (Sigma) and hybridized in the same buffer containing 50ng/mL heat denatured biotin-labeled probes (5'-TGCAACTTAGTAATGTGCAATA—3')at 68°C for 1 h. After hybridization, the membrane was washed twice in 2×saline sodium citrate (SSC) (1×SSC = 150 mM sodium chloride and 15 mM sodium citrate) with 0.1% sodium dodecyl sulfate (SDS) at room temperature for 15 min and in 0.1 x SSC with 0.1% SDS at 60°C for 30 min. The blots were exposed to X-ray film at -80°C for 48 h and then analysed by laser densitometry.

### Dual-source computed tomography

Mice CT examinations were performed on a dual-source spiral CT scanner (Siemens Definition Flash, Siemens Healthcare, Forchheim, Germany). The tube voltage and tube current settings were optimized independently for each X-ray source. Settings were chosen to split the overall dose equally between the 2 imaging chains. CT examinations were performed using the following protocol: Tube A, 80 kV; Tube B, 140 kV with tin (Sn) filter, 200 mAs and 77 mAs, respectively, and a collimation of 32×0.6 mm.

Patients CT examinations were performed on the same dual-source CT System as mice. All patients were instructed about breath holding and the importance of immobility during scanning. Just before the injection of contrast medium, 0.5–1.0 mg nitroglycerin was administered sublingually for vessel dilation. All scans were performed in a craniocaudal direction with prospective electrocardiogram (ECG)-triggering sequential protocol. The computed tomography coronary angiography (cCTA) scanning parameters were chosen individually for each patient depending on the BMI. The scanning parameters were as follows: range of data acquisition at 65–75% of the R-R interval when baseline heart rate (HR) was ≤70 bpm or 35–45% of the R-R interval when baseline HR was >70 bpm, slice collimation 128 × 0.6 mm with a z-flying focal spot, gantry rotation time 280 ms, reference tube current—time product 300 mAs, tube voltage 100 kV (BMI< 25 kg/m^2^) or 120 kV (BMI≥ 25 kg/m^2^).

The Hardplaques application of the Syngo Dual Energy software was used. For default setting, r was set to an empirical value of 1.81. For evaluation, only voxels with CT numbers between 50 and 1000 HU on the weighted average image were analyzed; the range of the smoothing filter was set to two. Two radiologists (5 and 13 years experience in cardiovascular radiology, respectively) independently visually analyzed the DE images in a randomized blinded fashion. After processing the DE data, vascular system was subjectively evaluated for the presence of abnormal calcium attenuation in arteries by using axial and sagittal images and a semiquantitative four-point scale in which 1 = none signs of abnormal calcium–like attenuation, 2 = equivocal attenuation changes, 3 = less pronounced, and 4 = distinct. Each observer was free to adjust window settings and software magnification.

### Luciferase reporter assay

The region of mouse PTEN-3'UTR (wild-type and mutant), generated by chemically synthesized, was cloned into the GV272 luciferase reporter plasmid (Genechem Co. Ltd., China). These constructs were named PTEN (NM008960-3'UTR) and PTEN (NM008960-3'UTR-mut). Forluciferase the reporter assay, MVSMCs were plated onto 12-well plates and transfected with 300 ng of PTEN-wt or PTEN-mut and 50 nM miR-32 mimics or 100 nM miR-32 inhibitors or their corresponding negative controls (RiboBio Co. Ltd., China) using lipofectamine 2000 (Invitrogen, Carlsbad, CA). After transfection for 48 h, cells were harvested and assayed with the Dual-Luciferase Reporter Assay System (Promega, San Luis Obispo, CA, USA). The tests were repeated in triplicate.

### Enzyme linked immunosorbent assay

The level of mice ALP and Runx2 in mice was determined by enzyme-linked immunosorbent Kit (Cusabio Bio Co.Ltd., China). In each well, 100μl standard samples were added and incubated for 2 hours at 37°C. The liquid of each well was then removed, and 100μl of indicated diluted Biotin-antibodies was added to each well. After incubating for 1 hour at 37°C, each well is aspirated and washed 3 times. Each well is then added 100μl of HRP-avidin (1×) and incubated for 1 hour at 37°C. After repeating the aspiration/wash process for five times, each well is added with 90μl of TMB Substrate and incubated for 15–30 min at 37°C, and then 50μl of Stop Solution was added to terminate the reaction.The reaction was subjected to Multiskan MK3 Microplate Reader (Thermo Fisher Scientific Inc, Waltham, MA USA) at the wavelength of 450 nm within 5 min.

### ALP activity determination

The ALP activities in the culture medium of miR-32 mimic transfected MVSMCs and in the plasma of patients with CAD were determined by using the colorimetric assay (Alkaline phosphatase assay kit, Nanjing Jiancheng Bioengineering Institute, China). Briefly, double-distilled water, Phenol standard solution, buffer solution and matrix solution were mixed with different samples andincubated for 15 minutes at 37°C. Chromogenic agent was then added to mixture and well mixed and ALP enzyme activity was measured at the wavelength of 520 nm by Elx808^TM^ Absorbance Microplate Reader (BioTek,Vermont, USA).

### Hematoxylin-eosin staining

The mice were received 4% chloral hydrate intraperitoneal anesthesia and the aorta were excised and cut into sections of 5-mm thickness. After embedding in paraffin, tissue sections from all the case were stained with hematoxylin and eosin (H&E, Sigma) for routine histological examination and detection of calcium deposition. The pictures of tissue sections were taken by Optical Microscope System (Olympus IX71, Olympus Corporation, Japan).

### microRNA microarray

To identify miRNAs involved in vascular calcification, we collected the aortas at the same location of mice vessels, and ground it in liquid nitrogen before miR extraction, andtotal RNA (2μg) from mice aortas was respectively labeled and manually hybridized to miRCURYTM LNA Array (v.18.0) (Exiqon, Vedbaek, Denmark) following the manufacturer’s protocol. Differential labeling of total RNA samples with dyes spectrally equivalent to Cy3TM and Cy5TM fluorophores allowed comparison of miRNAs expression levels of OPG^−/−^ and wild type mice. The labeling method allows selective labeling of miRNA in the total RNA sample. The hybridized microarrays were scanned with a GenePix 4000B instrument, and scanned images were then imported into GenePix Pro 6.0 software (Axon, Gilze, Netherlands) for grid alignment and data extraction. Replicated miRNAs were averaged and miRNAs that intensities≧30 in all samples were chosen for calculating normalization factor. Expressed data were normalized using the Median normalization. After normalization, significant differentially expressed miRNAs were identified through Volcano Plot filtering. Hierarchical clustering was performed using MEV software (v4.6, Tigr, La Jolla, CA, USA).

### Immunohistochemical staining

Paraffin-embedded aorta sections were deparaffinized in xylene, and rehydrated in ethanol. Deparaffinized sections underwent a microwave treatment in citrate buffer (10 mmol/L, pH 6.0) twice for 5 min, and rinsed in tap water to retrieve antigens. Endogenous peroxidase was inhibited with 0.3% H_2_O_2_ methanol for 10 min, followed by a rinse in phosphate buffered saline. Sections were blocked by 10% serum at room temperature for 30 min. For immunostaining of osteoblast-related proteins, such as ALP and Runx2 in the aortas, rabbit polyclonal anti-ALP (Abcam, Cambridge, UK) and mouse monoclonal anti-runx2 (Abcam, Cambridge, UK) antibodies were used as the primary antibodies respectively. The slides were incubated with the secondary antibody at room temperature for 30 min, and then with peroxidase-conjugated streptavidin (Nichirei Bio.Inc., Tokyo, Japan) at 37°C for 30 min. After another wash with PBS, peroxidase activity was detected by reaction with 3,3-diaminobenzidine tetrahydrochloride (DAB tablet, Merck KGaA, Darmstadt, Germany; 30 mg/mL, containing 0.03% H_2_O_2_) as the chromogen. Finally, sections were counterstained by 1% methylene green, dehydrated and mounted. Staining without the primary antibody was used as a negative control.

### Digital Subtraction Angiography (DSA)

A cardiointerventionalist performed conventional intra-arterial DSA studies by using a transradial artery approach and selective coronary artery catheterization with a digital angiography unit (Artis zee floor, Siemens Medical Solutions, Forchheim, Germany). Images of each vascular branch were obtained in both anterior-posterior and lateral views. Additional views were also added on an individual, to better visualize the coronary artery stenosis. At each branch, 7–8 ml of Iopromide 370 (Ultravist®, Schering AG, Berlin, Germany) was injected at a flow rate of 5 mL/sec. The DSA was performed with a 25 cm field of view, and a 1024×1024 matrix with spatial resolution of 0.25 mm.

### Clinical study

The clinical study, which complied with the Declaration of Helsinki, was approved by the ethics committee of the First Affiliated Hospital of University of South China (No.2012-07).All participants gave their written informed consent to participate in this study. The enrolled population was from a registry of CAD patients treated at First Affiliated Hospital of University of South China. The participants were recruited to the study since May 2013. We enrolled CAD patients between May 2013 and June 2014 at our Department of cardiovascular medicine, and we obtained information to identify individual participants. We excluded patients with established liver disease, cancer, and inflammatory disease which can affect ALP levels, prior to the procedure. Moreover, exclusion criteria were presence of stroke or acute myocardial infarction within the last 3 months, active malignancy, proliferative retinopathy, uncompensated heart failure, unstable angina, severe valvular disease, serious arrhythmia, severe rheumatologic disease, thromboembolic disease, chronic obstructive pulmonary disease, severe uncontrolled diabetes mellitus, severe musculoskeletal disorders, chronic hepatopathy, ongoing infections, digestive tract disorder, endocrine disorder, and patients with eGFR <60ml/min/1.73m^2^.As a result, 66 CAD patients were enrolled in this study. The CAD patients with CAC were determined by Digital Subtraction Angiography (DSA, GE Advantx LCV+, USA) and CT scanning(Siemens Definition Flash, Siemens Healthcare, Forchheim, Germany). According to the test results, patients are divided into CAC group (calcification group, including 33 samples) and non CAC group (control group, including 33 samples). Human peripheral blood from the patients was used for miRNA isolation by using miRcute miRNA Isolation Kit (Tiangen Bio. Co.Ltd.,China). To detect the plasma concentrationof miR-32-5p, we supplied poly(A) tail with 3’-end regions of miRNA, and do reverse transcription process by oligo(dT)-universal tag to get first-strand of cDNA (miRcute miRNA First-Strand cDNA Synthesis Kit, Tiangen, China). We detected plasma miR-32-5p using miRcute miRNAqPCR Detection kit (SYBR Green, Tiangen, China) according to the manufacturer’s guidelines. PCR reactions were incubated at 94°C for 2 min, followed by 40 cycles at 94°C for 20 seconds and 60°C for 34 seconds. All samples were assayed in triplicate, and data were normalized to circulating miRNA-16 (Tiangen Bio. Co.Ltd., China).

### Statistical analysis

All experiments were performed with three replicates and the results were expressed as the mean ± S.E.M. Student t test or One way ANOVAwith Bonferroni correctionwas used to assess the statistical significance. *P* values < 0.05 were considered significant. Wherein, since small number of animals used in the animal studies, we re-analyzed the related data with non-parametric statistical analyse method (Mann-whiteney U test). Correlation analysis was performed by Spearman's Rank Correlation test.

## Results

### Characteristics of vascular calcification in OPG^−/−^mice

**Fig 1:**
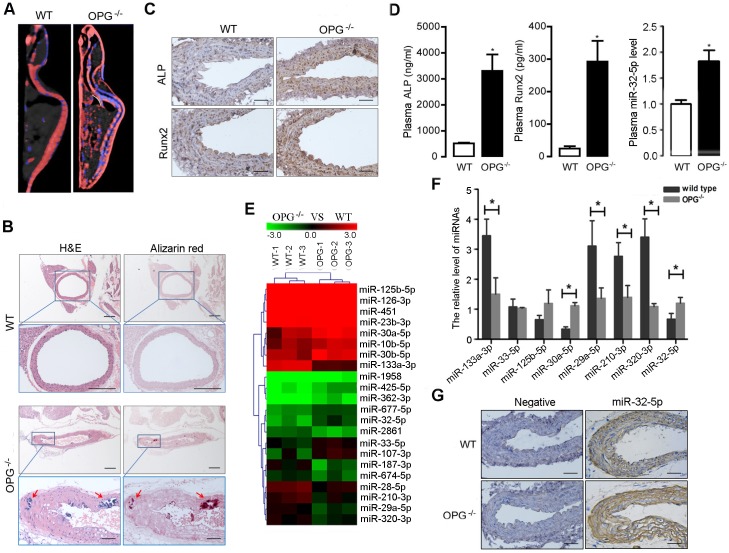
Characterizations of vascular calcification and identification of aortic calcification associated miRNAs of OPG deficient mice. **A**, The phenotype of OPG^-/-^ mice were determined by using Dual Source CT; OPG^-/-^ mice showed pronounced calcium loss in spine and obvious calcification in aorta, heart and internal organs at 12 weeks of age (n = 3). **B**,H&E(Hematoxylin and Eosin) staining and Alizarin Red staining showed that OPG^-/-^ mice exhibited obvious aortic calcification at 12 weeks after birth; arrows represent the calcified nodules (4×, scale length 200 μm) and corresponding magnified ones (20×, scale length 50 μm) in aortic artery (n = 3). The artery staining of correspondingwild type mice (WT)showed no obvious calcification (upper, 4×; lower, 10×. scale length 200 μm). **C**, The expression of ALP and Runx2 in aortic artery were determined by immunohistochemistry analysis in wild type and OPG^-/-^ mice at the age of 12 weeks (20×) (n = 3), scale length was 50 μm. **D**, The level of plasma ALP and Runx2 in mice was determined by enzyme-linked immunosorbent assay, and the level of plasma miR-32 was determined by real-time PCR. ALP, Runx2 and miR-32 showed an increased level in plasma of OPG^-/-^ mice at the ages of 12 weeks, compared to those in WT mice (n = 3). **E**, The cluster maps for the differentially expressed miRNAs; comparisons were made between OPG^-/-^ mice and WT mice at 12 weeks of age (n = 3). **F**, The differential expression miRNAs were confirmed with OPG^-/-^ mice and WT mice at 12 weeks of age by quantitative reverse transcription polymerase chain reaction (Q-PCR) (n = 3). **G**, The expression and distribution of miRNA-32 in aortic arteries of OPG^-/-^ and OPG^+/+^ mice were determined by *in situ* hybridization (20×), scale length was 50 μm. *P < 0.05, **P < 0.01 compared with WT mice group, data are shown as means±SEM and Mann-whiteney U testis used for comparison.

OPG^−/−^ mice develop severe medial calcifications of the renal arteries and aorta [9]. In this study, we determineddifferential miRNA-expression profiles during the progression of aortic calcification inanOPG^−/−^ mouse model. The calcification status of aortas fromOPG^–/–^and WT mice at different ages was determined by computed tomography (CT) scan and confirmed by pathological examination. As shown in Fig 1A, 12-week-old OPG^–/–^mice showed pronounced calcium loss in the spine and marked calcification in the aorta, heart, and internal organs. Additionally, hematoxylin-and-eosin and Alizarin Red staining showed that OPG^–/–^mice exhibitedclear evidence of aortic calcification (Fig 1B). Furthermore, expression of both ALP and RUNX2 increased significantly in the aortas of OPG^–/–^mice as compared with that observed in WT mice (Fig 1C), with levels of ALP and RUNX2also markedly higher in OPG^–/–^mice relative to levels in WT mice (Fig 1D).

### Identification of miRNAs associated with aortic calcification in OPG^–/–^mice

To determine the miRNAs involved in the aortic calcification process, we performed miRNA-microarray analysis(3100 probes) using total RNAs prepared from the following four groups: aortas without calcification of WTmice at 4 weeks of age, aortas without calcification of OPG^-/-^ mice at 4 weeks of age, aortas without calcification of OPG^+/+^ mice at 12 weeks of age, and aortas with calcification of OPG^-/-^ mice at 12 weeks of age. Comparisons were made among three groups: WTmice versus OPG^-/-^ mice at 4 weeks of age; WTmice versus OPG^-/-^ mice at 12 weeks of age; OPG^-/-^ mice at 4 weeks of age versus OPG^-/-^ mice at 12 weeks of age.Our results indicated thatmiRNAs exhibited differential expression in aortas between WT and OPG^–/–^mice (Fig 1E, S1–S3 Tables). Several miRNAsidentified as calcification modulators, such as miR-125b [17],exhibited a consistent increasein expression in the aortasofOPG^–/–^mice. Moreover, miR-32 was found to show 3.657 fold of down-regulation and 2.242 fold of up-regulation in OPG^-/-^ mice at the time point of 4-week-old and 12-week-old respectively, compared to those in WT mice, showing that miR-32 was the most changed and active miRNA during the vascular calcification progression. Given thatprevious studies of miR-32 focusedmainly on its role in cancer progression, includingcolorectal cancer [29],prostate cancer [30], andhepatocellular cancer [31], there are nocurrent studies available investigating the role ofmiR-32 in vascular calcification. Our results indicated that miR-32 might beanovel miRNA associated withthe progression of vascular calcification.

Based on the dynamic changes in miRNA-expression profiles associated with vascular calcification *in vivo*, eight miRNAs exhibiting obvious variationswere chosen for validation by quantitative PCR. Seven of the eight miRNA-expression profileswere validated as consistent with the trends observed from microarray analysis (Fig 1F), withmiR-32 confirmed as being elevated in the aortas of OPG^–/–^mice.*In situ* hybridization using paraffin sections of mouse aortas further validated the *in vivo* miR-32 levels and distribution. Interestingly, miR-32 localized mainly in the tunica media of aortas and exhibitedelevatedlevels in aortas fromOPG^–/–^mice as compared withlevels observed inaortas from WT mice (Fig 1G). These results indicated that elevatedmiR-32 expression might be involved in vascular calcification as a potential modulator of calcification progression.

### MiRNA expression during osteogenic differentiation of mice VSMCs

**Fig 2:**
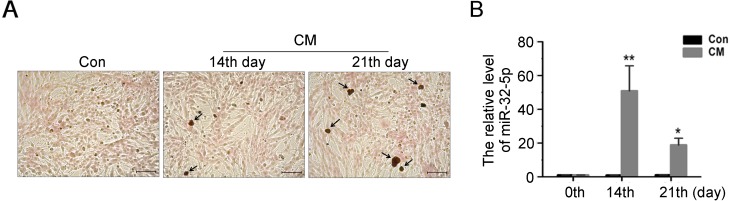
The miRNAs expression during osteogenic differentiation of MVSMCs. **A**, MVSMCs were cultured in DMEM containing CM (calcification medium) for 14 and 21 days, and cells were stained with Alizarin red for the detection of calcification nodules (20×), scale length was 50 μm. **B**, The mRNA level of miR-32expression in CM-treated MVMSCs were detected by Q-PCR, and data are shown as mean±SEM of experiments performed in triplicate (n = 3). *P< 0.05, **P < 0.01 versus control(Con).

To verify miR-32 involvement in vascular calcification *in vivo* and *in vitro*, we established a cellular calcification model using MVSMCs treated with CM. MVSMCs were maintained in CM for 21 days anddeveloped calcification nodules at 14 and 21 days (Fig 2A).

MiR-32 expression was determined by quantitative PCR to confirm its role during MVSMC osteogenic differentiation. Our results indicated that miR-32 expression exhibited a significant increase at day 14, whichwas sustainedthroughday 21, compared with MVSMC cultured with control medium for relative corresponding days (Fig 2B). These data confirmed a potential role for miR-32 in vascular calcification.

### MiR-32 promotes MVSMC calcification by inducing expression of vascular calcification markers

**Fig 3:**
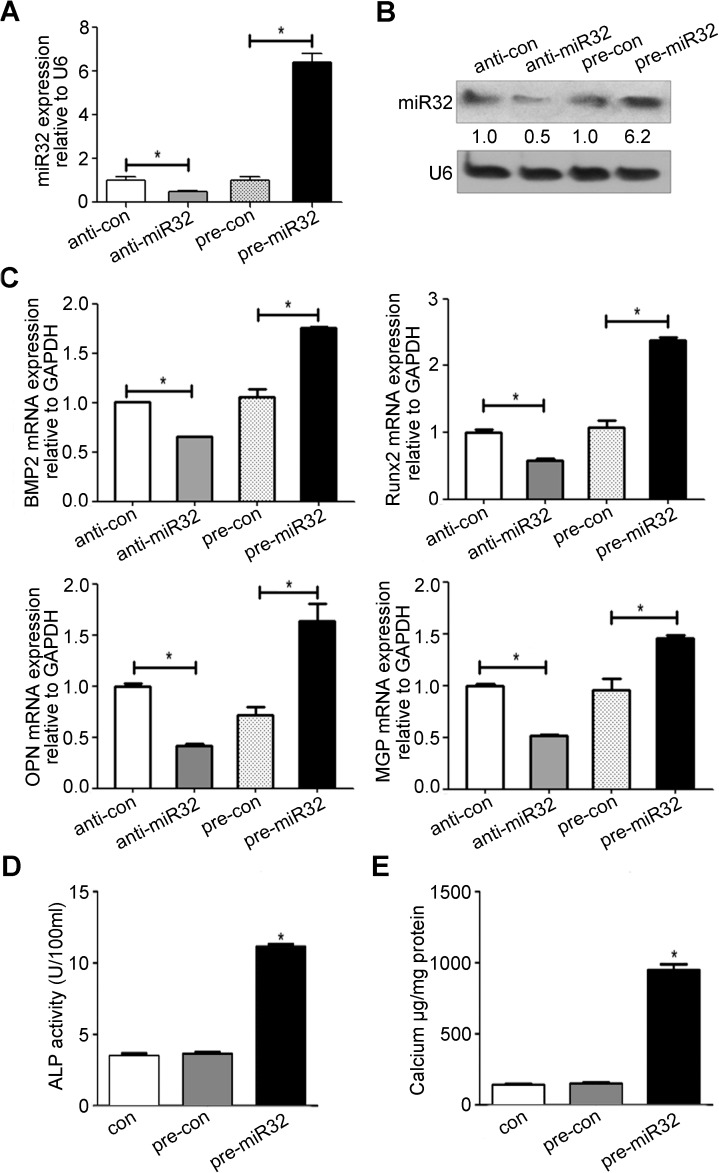
MiR-32 induces the expression of vascular calcification markers. **A&B**, The miR-32 mimics(pre-miR32),negative control of miR-32 mimics (pre-con), miR-32 inhibitors (anti-miR32), or negative control of miR-32 inhibitors (anti-con) were transfected into MVMSCs for 4 days and miR-32 expression level was determined by Q-PCR (**A**) and Northern Blot (**B**) (n = 3). **C**, The effect of miR-32 on the BMP2, Runx2, OPN and MGP mRNA level in MVSMCs were detected by Q-PCR at the time point of 4 days after the transfection of miR-32 mimics and miR-32 inhibitorsin MVMSCs (n = 3). **D&E**, The effect of miR-32 on ALP activity and calcium content of MVSMCs were also determined at the time point of 4 days after miR-32 mimics transfection(n = 3). Data are shown as mean±SEM of a representative experiment performed in triplicate, *P< 0.05, ** *P*< 0.01.

To determine the extent of miR-32 involvement in vascular calcification, miR-32 mimic or anti-miR-32 was transfected into MVSMCs, and the transfection efficiency was confirmed by quantitative PCR and northern blot. As shown in Fig 3A and 3B, transfection with the miR-32 mimic markedly increasedmiR-32 levelsin MVSMCs on the 4th daypost-transfection, whereastransfection with anti-miR-32 resulted indecreasesin miR-32 levels. We then determined whether miR-32 affectsthe progression of MVSMCs calcification. We observedthat cells transfected with the miR-32 mimic or anti-miR-32 inducedsignificant increasesor decreasesinBMP2, RUNX2, OPN, and MGPmRNA levelsrespectively,4 dayspost-transfection(Fig 3C). The effect of miR-32 on ALP activity was also determined, revealingincreases in ALP activity in MVSMCs transfected with the miR-32 mimic(Fig 3D).The effect of miR-32 on MVSMC calcium content was also determined, revealing that ectopic miR-32 expression resultedin marked increasesin calcium content(Fig 3E). Given our observation of the altered expression of calcification markers, including BMP2, RUNX2, OPN, MGP, and ALP,these resultssuggested that miR-32 might function as a modulatorof MVSMC calcification.

### MiR-32 enhances RUNX2 expression and activity

**Fig 4:**
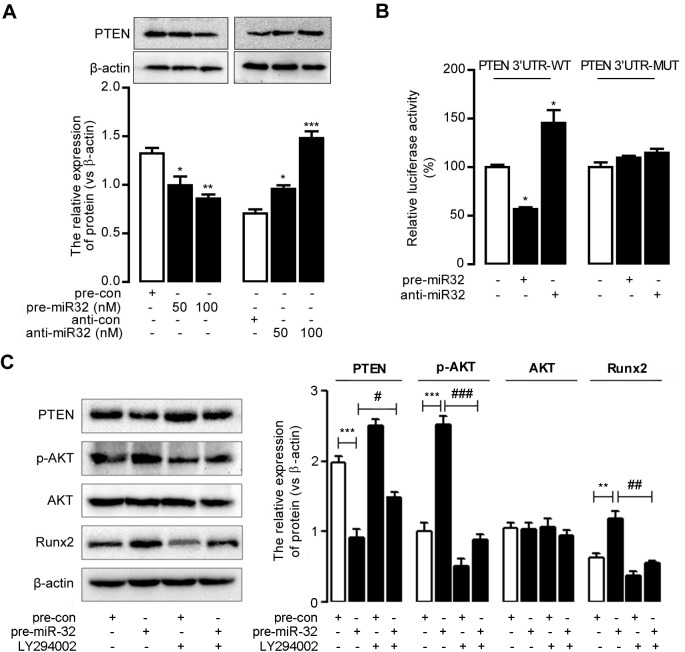
MiR-32 activates PI3K-Akt pathway by targeting toPTENand enhances Runx2 expression and phosphorylation. **A**, The miR-32 mimics (pre-miR32),negative control of miR-32 mimics (pre-con), miR-32 inhibitors (anti-miR32), or negative control of miR-32 inhibitors (anti-con)were transfected into MVSMCs and the cells were lysed and analyzed by immunobloting with the indicated antibody. *P< 0.05, **P< 0.01, ***P< 0.001, compared to control groups. **B**, Effect of miR-32 on PTEN expression by luciferase reporter assay. MVSMCs were cotransfected with pre-miR32 (or anti-miR32) and PTEN 3’UTR wild-type vectors (PTEN 3’UTR-WT) or PTEN 3’UTR mutant vectors (PTEN 3’UTR-MUT). Luciferase activity was normalized by the ratio of firefly and renilla luciferase signals. **C**, PI3K signaling is involved in miR-32 induced Runx2 expression and phosphorylation. MVSMCs were transfected with indicated concentration of miR-32 mimics or pre-con, with or without LY294002 and total protein extracts were analyzed by Western blot.Data of panel **A**are analyzed with ANOVA with Bonferroni correction, and student t test is used for data anaysis of panel **B**&**C**. *P< 0.05,**P< 0.01.

MiR-32 activated sphosphoinositide 3-kinase (PI3K) signaling in bone marrow-derived mesenchymal stem cells [28], colorectal cancer cells [29], and hepatocellular cancer cells [31] by suppressing PTEN levels via targeting the 3′ untranslated region (UTR)of PTEN mRNA. We observed that miR-32 reducedPTEN expression in MVSMCs, which was detected by western blot (Fig 4A). Luciferase-reporter assay results confirmed the miR-32-mediateddownregulation PTEN via 3′-UTR targeting (Fig 4B).

RUNX2is an essential modulator of osteoblast differentiation and vascular calcification [32]; therefore,we determined whether miR-32 affects RUNX2 expression and phosphorylation through PI3K-Aktsignaling. As shown in Fig 4C, ectopic expression of miR-32 inducedAkt phosphorylation, resulting inincreased RUNX2 expression. Inhibition of Akt phosphorylation abrogated miR-32-induced RUNX2 expression, indicating the involvement of Akt signaling in miR-32-mediatedRunx2 activity in MVSMCs. These results suggested that miR-32 might promote cellular calcification by increasing RUNX2 expression bydecreasing PTEN levels and subsequently activating the PI3K-Akt-signaling pathway in MVSMCs.

### MiR-32 is upregulated in plasmafrom patients exhibiting coronary artery calcification(CAC)

**Fig 5:**
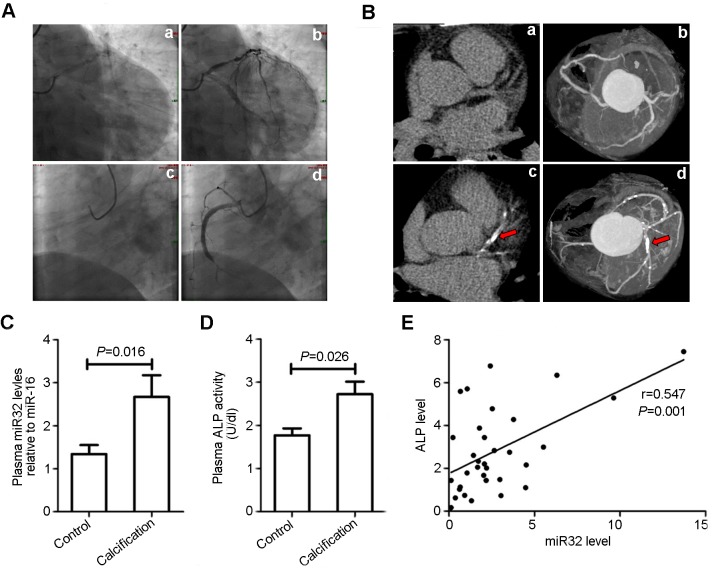
MiR-32 shows increased level in plasma of patients with coronary artery calcification. **A**,The representative images of aortic stenosis of CADpatients confirmed by coronary arteriongraphy (n = 66). a&c (two pictures upper) represent the coronary artery before coronary arteriongraphy and b&d (two pictures lower) represent the coronary artery after coronary arteriongraphy; a&b, CAD patients without coronary calcification; c&d, CAD with calcification.**B**,Representatives of transverse (a&c, two left pictures) and maximum intensity projection (b&d, two right pictures) images of coronary arteries by CT scanning; a&b, examples of no coronary calcium; c&d, examples of coronary calcification; arrows indicate the calcification of coronary artery.**C**,Plasma from 66 patients with coronary heart disease of which 33 patients showed calcification in coronary artery confirmed by CT scanning were collected for miRNA-32 detection by Q-PCR. Data are shown as mean±SEM of experiments. **D**, The plasmaALP activity of patients was detected as described in methods, and data are shown as mean±SEM of experiments (n = 66). **E**,the scattering spots image showed the positive correlation between miRNA-32 and ALP activity in plasma of patients with coronary artery calcification (n = 33), which was performed by Spearman's Rank Correlation test.

MiRNAs constitute a novel class of blood-based biomarkers, the expression of which can be associated with disease development and severity [33]. Therefore,we detectedmiR-32 levels in plasmaextracted fromCAD patients exhibitingCAC.Plasma from 66 CAD patients, 33 of which showed calcification in the coronary artery as confirmed by CT scanning (Fig 5A),was collected, andmiR-32 levels were assessedby quantitativePCR. Our results revealed that miR-32 levelsexhibited marked increases in plasmafrom CAD patients with CACas compared with levels measured in patients without CAC (*P* = 0.016) (Fig 5B). Additionally, detection ofALP activity in plasma from CAD patients revealedhigher ALP activity in CAD patients with CAC as compared with ALP activity measured inpatients without CAC (*P* = 0.026) (Fig 5C). Interestingly, we observed a positive correlation between miR-32 and ALP activity in CAD patients between the ages of 50 and 80 with CAC (*r* = 0.547;*P* = 0.001) (Fig 5D), which was consistent with levels of miR-32-induced ALP activity in MVSMCs (Fig 3D). These clinical data supporteda potential role for plasma miR-32 as a diagnostic marker in CAD patients with CAC. However, further studies involving a larger sample size is needed to validate these results.

## Discussion

Highly complex mechanisms continue to be identified involving the initiation and progression of vascular calcification in various human pathologies, including atherosclerosis, diabetes mellitus, and chronic kidney disease. Researchers also embrace the concept that vascular calcification is an actively regulated process rather than one involving passive calcium deposition and degeneration. Although various molecular mechanisms, including abnormal calcium or phosphate metabolism, osteogenic reprogramming of VSMCs, apoptosis, oxidative stress, inflammation, and loss of mineralization inhibitors, have been delineated in the process of vascular calcification [34,35], the understanding ofthe associated mechanisms is far from comprehensive, and an effective preventive or therapeutic strategy for vascular calcification remains unidentified. Here, we identified miRNA-expression profiles related to the progression of vascular calcificationand provided evidence for the involvement of miR-32 in VSMC calcification.

OPG inhibits osteoclast formation when administered *in vivo* to mice or when added to an *in vitro* osteoclast-forming assay [7].OPG^−/−^ mice exhibit late medial calcification of the renal and aortic arteries [8,9], with intima and media calcification classified by different distribution sites of pathological calcium-phosphate deposition in the arterial wall. In this study, we used OPG^−/−^ mice as a calcification model to determinemiRNAs involved in the progression of aortic calcification. Our resultsidentified 20differentially expressed miRNAs in aortas from WT mice and 12-week-old OPG^−/−^ mice, with miR-32, miR-125b, miR-30a, miR-29a, miR-210, miR-33, miR-133a exhibitingsignificantly altered expression in aortas fromOPG^−/−^ mice relative to their expression in aortas from WT mice. Because miR-133a, miR-125b, miR-29a, and miR-210 werepreviously implicated in the progression of vascular calcification [13,17,36,37],we were able to confirm the validity of OPG^−/−^ mice as suitable models for the screening of miRNA profiles associated with vascular calcification. Moreover, the differential expression of miR-32 indicated a possible novelrole in calcification modulation in addition to its reported involvement in cancer progression [29–31].To determine the role of miR-32 in vascular calcification *in vitro* and *in vivo*, we developed a CM-induced cellular-calcification model using MVSMCs, with our results suggesting that miR-32 performs a pivotal function in the initiation and progression of vascular calcification.

We performed *in situ* hybridization to explore the distribution and expression of miR-32 in aortas, revealing its localization mainly in the media and confirming its increased expression in the aortas of OPG^−/−^ mice as compared with levels observed in WT mice. Additionally, we used *in vitro* MVSMC-calcification models in the presence of CM to investigatemiR-32-induced alterations of calcification development.Our results confirmed that miR-32 levelswere elevated atculture day 14 and remained high throughday 21, confirming that miR-32 exhibited increased expression during *in vitro* and *in vivo* osteogenic differentiation. Furthermore, we revealed thatforced expression of miR-32 resulted in elevatedexpression of BMP2, RUNX2, OPN, and MGP, as well as increased ALP activity, in MVSMCs, whereas miR-32 downregulation subsequently attenuated the increases in these cellular markers during calcification progression. These results suggested that miR-32 might function as a positive modulator of cellular calcification.

As a typical pathway, PI3K signaling pathway has been discussed well in vascular calcification. For its positive role, activation of PI3K/Akt signaling by O-Linked N-Acetylglucosamine was varified to induce vascular calcification in Diabetes Mellitus [38], and PI3K/Akt signaling was found to promote oxidative stress-induced VSMC calcification [32]. Otherwise, for its negative role, inhibition of PI3K or Akt activation reversed the effects of omentin on ALP activity and the matrix mineralization [39], and Gas6/Axl-PI3K/Akt pathway was found to inhibited inorganic phosphate-induced calcification of vascular smooth muscle cells by statins administration [40]. It was clear that miR32 could target PTEN to affect multiple carcinoma progress, such as hepatocellular carcinoma and colorectal cancer [29,31], andmiR-32 activatesPI3K signaling by targeting PTEN in bone marrow-derived mesenchymal stem cells [28]. As for PTEN, Liang Deng et al reported that PTEN deletion resulted in sustained activation of AKT that upregulated Runx2 and promoted VSMC calcification *in vitro* and arterial calcification ex vivo [41]. PTEN was also verified to be a key target of miRNAs in osteoclast differentiation [42]. Furthermore,miR-32 is involved in tumorigenesis viasuppression of PTEN expression by directly targeting the 3′-UTR of PTENmRNA [29,31], indicating that PI3K signaling could be a downstream target of miR-32-related activity. Here, we assessed the effect of miR-32 on PI3K signaling in MVSMCsand discovered that miR-32 enhancedAkt phosphorylation and suppressed PTEN expression in MVSMCs, indicating a potential role for the miR-32/PTEN/Akt axis in MVSMCs.

RUNX2 expression and phosphorylation is involved in osteoblast differentiation and vascular calcification and is essential and sufficient for driving smooth muscle cell calcification [32,43,44].Several calcification-related miRNAs regulate RUNX2 expression. Cui *et al*[20] reportedthat miR-204 inhibited VSMC calcification by targeting RUNX2, and Balderman *et al*[45] confirmed that miR-30b-c also influenced vascular calcification by regulating RUNX2. In this study, we observed that RUNX2 expression and phosphorylation were elevated by ectopic miR-32 expression. Our findings indicated that miR-32 might promote cellular calcification at least partly through modulating increases in RUNX2 expression and phosphorylation by directly targeting PTEN expression and activating PI3K signaling. Because miR-32 might have additional downstream targets involved in cellular calcification, our future workinvolveselucidation of the detailed mechanisms underlying miR-32-induced progression of cellular calcification.

MiRNAs have emerged as a novel class of biomarkers associated with disease development and severity, as well as response to treatment, with specific roles in a large spectrum of cancers [33]. To our knowledge, there areno reportsoutlining the existence of miR-32 in plasma; therefore, we detected miR-32levelsinplasma to investigatepossible associations between plasma miR-32 levels and vascular calcification. Interestingly, we observed higher plasmamiR-32 levels in CAD patients between the ages of 50 and 59 with CAC as compared with levels observed in similarly aged patientswithout CAC, which was consistent with levels observed in cell and animal models. ALP plays essential roles in mineral metabolism[46] and is a marker of coronary artery calcification [47]. Additionally, plasma ALP activity is an independent predictor of mortality, myocardial infarction,and stent thrombosis in CAD patients following percutaneous coronary intervention with a drug-eluting stent [48].Here, we determined a positive correlation between plasmamiR-32 levels and ALP activity in plasma from CAD patients with CAC, which was consistent with our observation that ectopic miR-32 expression increasedALP activity in MVSMCs. Thesefindings indicated that miR-32 could function as a modulator of ALP expression and activity and might constitute a potential biological marker for the diagnosis and prognosis of coronary artery calcification; however,a larger cohort for aclinical study is needed to confirm our results.

To our knowledge, this is the first report of dynamic miRNA-expression profiles related to the progression of vascular calcification *in vivo*.Our results confirmed miR-32 as a critical modulator of vascular calcification through its targeting of the PTEN/Akt/RUNX2 axis in mice. Moreover, results of analyses of plasmafrom CAD patients revealed that plasma miR-32 levels were positivelycorrelated withCAC and might serve as a diagnostic markerin CAD patients with CAC.These findingsenhanced the understanding of miRNA rolesin the progression of vascular calcification and provided insight intothe possible prevention and treatment of diseases associated with vascular calcification.

## Supporting information

S1 File Microarray analysis profiling data for miRNA expression. (XLS)

S1 Table Differentially expressedmiRNAs in aortic tissues of OPG^-/-^ mice compared with those in wild-type mice at4weeks of age. (DOCX)

S2 Table Differentially expressed miRNAs in aortic tissues of OPG-/- mice compared with those in wild-type mice at 12 weeks of age. (DOCX)

S3 Table Differentially expressedmiRNAs in aortic tissues of OPG^-/-^ mice at 12 weeks of age compared with those in OPG-/- mice at 4 weeks of age. (DOCX)

S4 Table Primers for Real Time PCR. (DOCX)
