# Supplementary material for: MicroRNA-32 promotes calcification in vascular smooth muscle cells: Implications as a novel marker for coronary artery calcification
Source: PLoS One. 2017 Mar 20;12(3):e0174138. doi: 10.1371/journal.pone.0174138 (PMC5358880; doi:10.1371/journal.pone.0174138)
Supplement: S1 Table — (DOCX) [file pone.0174138.s002.docx]

**S1 Table.** **Differentially expressed miRNAs in aortic tissues of OPG^-/-^ mice compared with those in wild-type mice at 4 weeks of age（part of miRNA data were shown）(n=3)**

| **Up-regulated** | | | | | | | | |
| --- | --- | --- | --- | --- | --- | --- | --- | --- |
| Accession | ID | | | Fold change | | | P value | |
| MIMAT0000138 | | | mmu-miR-126-3p | | | 3.521 | | 0.092 |
| MIMAT0000216 | | | mmu-miR-187-3p | | | 3.132 | | 0.070 |
| MIMAT0000208 | | | mmu-miR-10b-5p | | | 3.055 | | 0.110 |
| MIMAT0003740 | | | mmu-miR-674-5p | | | 2.047 | | 0.222 |
| MIMAT0000130 | | | mmu-miR-30b-5p | | | 2.046 | | 0.062 |
| MIMAT0000136 | | | mmu-miR-125b-5p | | | 1.729 | | 0.181 |
| MIMAT0004684 | | | mmu-miR-362-3p | | | 1.808 | | 0.222 |
| MIMAT0000647 | | | mmu-miR-107-3p | | | 1.598 | | 0.345 |
| MIMAT0000151 | | | mmu-miR-140-5p | | | 3.937 | | 0.035 |
| MIMAT0001632 | | | mmu-miR-451-3p | | | 3.322 | | 0.029 |
| MIMAT0004527 | | | mmu-miR-124-5p | | | 1.650 | | 0.031 |
|  |  | | | |  | |  | |
| **Down-regulated** | | | | | | | | |
| MIMAT0019339 | | mmu-miR-28-5p/mmu-miR-28c | | | | 1.672 | | 0.377 |
| MIMAT0000666 | | mmu-miR-320-3p | | | | 2.193 | | 0.169 |
| MIMAT0000654 | | mmu-miR-32-5p | | | | 3.675 | | 0.169 |
| MIMAT0013803 | | mmu-miR-2861 | | | | 8.416 | | 0.152 |
| MIMAT0017014 | | mmu-miR-208a-5p | | | | 3.354 | | 0.033 |
| MIMAT0003743 | | mmu-miR-712-3p | | | | 2.388 | | 0.035 |
