# Supplementary material for: MicroRNA-32 promotes calcification in vascular smooth muscle cells: Implications as a novel marker for coronary artery calcification
Source: PLoS One. 2017 Mar 20;12(3):e0174138. doi: 10.1371/journal.pone.0174138 (PMC5358880; doi:10.1371/journal.pone.0174138)
Supplement: S2 Table — (DOCX) [file pone.0174138.s003.docx]

**S2 Table. Differentially expressed miRNAs in aortic tissues of OPG^-/-^ mice compared with those in wild-type mice at 12 weeks of age (part of miRNA data were shown, n=3)**

| **Up-regulated** | | | | | | |
| --- | --- | --- | --- | --- | --- | --- |
| Accession | ID | | Fold change | | P value | |
| MIMAT0009431 | | mmu-miR-1958 | | 2.523 | | 0.033 |
| MIMAT0000654 | | mmu-miR-32-5p | | 2.242 | | 0.026 |
| MIMAT0000647 | | mmu-miR-107-3p | | 2.199 | | 0.043 |
| MIMAT0004684 | | mmu-miR-362-3p | | 2.109 | | 0.039 |
| MIMAT0004750 | | mmu-miR-425-5p | | 2.090 | | 0.029 |
| MIMAT0000136 | | mmu-miR-125b-5p | | 2.062 | | 0.018 |
| MIMAT0000128 | | mmu-miR-30a-5p | | 2.031 | | 0.019 |
| MIMAT0000130 | | mmu-miR-30b-5p | | 1.773 | | 0.035 |
| MIMAT0003451 | | mmu-miR-677-5p | | 1.764 | | 0.0003 |
| MIMAT0001632 | | mmu-miR-451-3p | | 1.660 | | 0.026 |
| MIMAT0000208 | | mmu-miR-10b-5p | | 1.649 | | 0.041 |
| MIMAT0000125 | | mmu-miR-23b-3p | | 1.646 | | 0.011 |
| MIMAT0000138 | | mmu-miR-126-3p | | 1.638 | | 0.006 |
| MIMAT0000667 | | mmu-miR-33-5p | | 1.621 | | 0.024 |
| **Down-regulated** | | | | | | |
| MIMAT0000145 | | mmu-miR-133a-3p | | 3.263 | | 0.000 |
| MIMAT0000216 | | mmu-miR-187-3p | | 2.596 | | 0.015 |
| MIMAT0013803 | | mmu-miR-2861 | | 1.883 | | 0.002 |
| MIMAT0004631 | | mmu-miR-29a-5p | | 1.797 | | 0.010 |
| MIMAT0000658 | | mmu-miR-210-3p | | 1.660 | | 0.044 |
| MIMAT0019339 | | mmu-miR-28-5p/mmu-miR-28c | | 1.633 | | 0.014 |
| MIMAT0003740 | | mmu-miR-674-5p | | 1.555 | | 0.063 |
| MIMAT0000666 | | mmu-miR-320-3p | | 1.511 | | 0.040 |
