# Supplementary material for: MicroRNA-32 promotes calcification in vascular smooth muscle cells: Implications as a novel marker for coronary artery calcification
Source: PLoS One. 2017 Mar 20;12(3):e0174138. doi: 10.1371/journal.pone.0174138 (PMC5358880; doi:10.1371/journal.pone.0174138)
Supplement: S3 Table — (DOCX) [file pone.0174138.s004.docx]

**S3 Table. Differentially expressed miRNAs in aortic tissues of OPG^-/-^ mice at 12 weeks of age compared with those in OPG-/- mice at 4 weeks of age（part of miRNA data were shown）(n=3)**

| **Up-regulated** | | | | | | | |
| --- | --- | --- | --- | --- | --- | --- | --- |
| Accession | ID | | Fold change | | P value | | |
| MIMAT0000654 | | mmu-miR-32-5p | | 3.953 | | 0.011 | |
| MIMAT0004631 | | mmu-miR-29a-5p | | 3.522 | | 0.004 | |
| MIMAT0000128 | | mmu-miR-30a-5p | | 3.402 | | 0.002 | |
| MIMAT0000667 | | mmu-miR-33-5p | | 2.756 | | 0.007 | |
| MIMAT0000658 | | mmu-miR-210-3p | | 2.541 | | 0.025 | |
| MIMAT0000130 | | mmu-miR-30b-5p | | 2.186 | | 0.012 | |
| MIMAT0003451 | | mmu-miR-677-5p | | 2.017 | | 0.005 | |
| MIMAT0000138 | | mmu-miR-126-3p | | 1.898 | | 0.016 | |
| MIMAT0004750 | | mmu-miR-425-5p | | 1.886 | | 0.034 | |
| MIMAT0000136 | | mmu-miR-125b-5p | | 1.533 | | 0.084 | |
| **Down-regulated** | | | | | | |  |
| MIMAT0017079 | | mmu-miR-377-5p | | 7.563 | | 0.018 |  |
| MIMAT0017209 | | mmu-miR-541-3p | | 5.720 | | 0.005 |  |
| MIMAT0017062 | | mmu-miR-224-3p | | 5.436 | | 0.000 |  |
| MIMAT0014815 | | mmu-miR-3099-5p | | 4.816 | | 0.000 |  |
| MIMAT0007870 | | mmu-miR-1900 | | 4.738 | | 0.003 |  |
| MIMAT0004839 | | mmu-miR-743b-5p | | 4.705 | | 0.007 |  |
| MIMAT0017060 | | mmu-miR-221-5p | | 3.210 | | 0.038 |  |
| MIMAT0017002 | | mmu-miR-204-3p | | 2.532 | | 0.009 |  |
| MIMAT0004628 | | mmu-miR-21-3p | | 2.197 | | 0.046 |  |
| MIMAT0004524 | | mmu-miR-30b-3p | | 1.634 | | 0.030 |  |
