# Supplementary material for: MicroRNA-32 promotes calcification in vascular smooth muscle cells: Implications as a novel marker for coronary artery calcification
Source: PLoS One. 2017 Mar 20;12(3):e0174138. doi: 10.1371/journal.pone.0174138 (PMC5358880; doi:10.1371/journal.pone.0174138)
Supplement: S4 Table — (DOCX) [file pone.0174138.s005.docx]

**S4 Table. Primers for Real Time PCR**

| Gene | Sequence | |
| --- | --- | --- |
| mmu-miR-32-5p | Sense | GGGCTATGGCACATTACTA |
|  | Antisense | CAGTGCGTGTCGTGGA |
| mmu-miR-133a-3p | Sense | GGTTTGGTCCCCTTCA |
|  | Antisense | CAGTGCGTGTCGTGGA |
| mmu-miR-125b-5P | Sense | CACTCCCTGAGACCCTAAC |
|  | Antisense | TGCGTGTCGTGGAGTC |
| mmu-miR-320-3p | Sense | GGAAAAGGTGGGTTGAGA |
|  | Antisense | CAGTGCGTGTCGTGGAG |
| mmu-U6 | Sense | GCTTCGGCAGCACATATACTAAAAT |
|  | Antisense | CGCTTCACGAATTTGCGTGTCAT |
| hsa-miR-32-5p | Sense | CD202-0093 , TIANGEN |
| hsa-miR-16-5p | Sense | CD202-0007 , TIANGEN |
| Drosha | Sense | CAATGATGCTGCCTGTTCT |
|  | Antisense | TTCTGTCTCCATATTTGGGTT |
| Dicer | Sense | AGCCGTCAGAACTCACTCG |
|  | Antisense | GAATCGCAAGATGACACTC |
| GAPDH | Sense | GAGACCTTCAACACCCCAGC |
|  | Antisense | ATGTCACGCACGATTTCCC |
| Runx2 | Sense | AACTTCCTGTGCTCCGTGCT |
|  | Antisense | CCTGGCTACTTGGTTTTTCA |
| MGP | Sense | CACCCGAGACACCATGAAGAG |
|  | Antisense | GCAGCGTTGTAGCCGTAGACC |
| BMP2 | Sense | TGCCCCCCTATATGCTAGAT |
|  | Antisense | GCAGATGTGAGAAACTCGT |
| OPN | Sense | GAATCTGACGAATCTCACCAT |
|  | Antisense | ACTCCTTAGACTCACCGCTCT |
